# Comparing microbiota profiles in induced and spontaneous sputum samples in COPD patients

**DOI:** 10.1186/s12931-017-0645-3

**Published:** 2017-08-29

**Authors:** Solveig Tangedal, Marianne Aanerud, Rune Grønseth, Christine Drengenes, Harald G. Wiker, Per S. Bakke, Tomas M. Eagan

**Affiliations:** 1 Department of Thoracic Medicine, Haukeland University Hospital, Bergen, Norway; 2 Department of Clinical Science, Faculty of Medicine and Dentistry, University of Bergen, Bergen, Norway; 3 Department of Microbiology, Haukeland University Hospital, Bergen, Norway

**Keywords:** COPD, Sputum, Microbiota, High-throughput sequencing

## Abstract

**Background:**

Induced and spontaneous sputum are used to evaluate the airways microbiota. Whether the sputum types can be used interchangeably in microbiota research is unknown. Our aim was to compare microbiota in induced and spontaneous sputum from COPD patients sampled during the same consultation.

**Methods:**

COPD patients from Bergen, Norway, were followed between 2006/2010, examined during the stable state and exacerbations. 30 patients delivered 36 sample pairs. DNA was extracted by enzymatic and mechanical lysis methods. The V3-V4 region of the 16S rRNA gene was PCR-amplified and prepared for paired-end sequencing. Illumina Miseq System was used for sequencing, and Quantitative Insights Into Microbial Ecology (QIIME) and Stata were used for bioinformatics and statistical analyses.

**Results:**

Approximately 4 million sequences were sorted into 1004 different OTUs and further assigned to 106 different taxa. Pair-wise comparison of both taxonomic composition and beta-diversity revealed significant differences in one or both parameters in 1/3 of sample pairs. Alpha-diversity did not differ. Comparing abundances for each taxa identified, showed statistically significant differences between the mean abundances in induced versus spontaneous samples for 15 taxa when disease state was considered. This included potential pathogens like *Haemophilus* and *Moraxella.*

**Conclusion:**

When studying microbiota in sputum samples one should take into consideration how samples are collected and avoid the usage of both induced and spontaneous sputum in the same study.

## Background

Chronic obstructive pulmonary disease (COPD) is characterized by a chronic inflammation of the lower airways, dominated by an influx of innate immune cells. Recent marker-gene studies indicate the existence of a pulmonary microbial flora (microbiota) present in both health and disease [1]. The chronic inflammation seen in COPD might be a consequence of a disrupted equilibrium between the pulmonary microbiota and the innate immune system. To explore this hypothesis, accurate measurements of the microbiota during both stable state and acute exacerbation of chronic obstructive pulmonary disease (AECOPD) are necessary.

The emerging gold standard for exploring the microbiota in the lower airways with minimal oral contamination is through bronchoscopy, but this is impossible during most AECOPD. Collecting induced sputum samples (ISS) is therefore a standardized sampling method of choice [2]. However, in several studies spontaneous sputum samples (SSS) have also been used since they are easier to retrieve [3, 4]. The validity of SSS with regard to microbiota studies is uncertain to date. Two previous studies have compared the microbial composition in ISS and SSS samples from cystic fibrosis (CF) patients, finding comparable results between the two methods [5, 6]. However, CF patients usually produce more sputum spontaneously, have a relatively high biomass in the airways, and one of the cited studies used an earlier method of bacterial profiling (Terminal Restriction Fragment Length Polymorphism Profiling) [6], wheras the other had only 15 sputum pairs [5]. The validity of SSS with regard to 16S rRNA marker-gene based studies on non-CF patients is unknown to date.

The Bergen COPD Cohort Study (BCCS) and its adjunct Bergen COPD Exacerbation Study (BCES) offers an opportunity to address this issue in COPD patients as we have sampled sputum both induced and spontaneously in a number of our COPD patients repeatedly during follow-up. We have previously shown that levels of inflammatory markers differed between sputum types in a study from the same population [7]. In the present study we compared the taxonomic composition and diversity measures in 36 sputum pairs consisting of SSS and ISS sampled sequencially from COPD patients either during AECOPD or at the stable state.

## Methods

### Study design

The current study sample consisted of 36 sputum pairs collected from 30 COPD patients who participated in both the BCCS & BCES. The study design and sampling of the BCCS [8] and the BCES [7] has previously been described in detail. The COPD patients had a smoking history of ≥10 pack-years, and a post-bronchodilation FEV_1_/FVC ratio < 0.7 and FEV_1_ < 80% predicted. Active autoimmune diseases or cancer within the last 5 years were cause for non-inclusion. A study physician examined and undertook a structured interview of all patients upon inclusion and at half-yearly follow-up visits when the patients were in the stable state.

Patients were instructed to contact the study-staff at periods with worsening of symptoms (malaise, fever, airway symptoms). The study physician offered a clinical examination at the outpatient clinic, Dept. of Thoracic Medicine, Haukeland University Hospital within 24 h of contact, or on the first working day after the weekend. Hospitalized patients were examined by a study physician the first day after admission.

The study was approved by the regional ethical board (REK-Vest), case number 165.08.

### Sputum sampling and processing

Both sputum sampling and immediate processing have been described in detail [7]. SSS was collected first from patients expectorating. If the patient’s clinical state allowed it, induction with hypertonic saline (3%) was performed. Patients inhaled the saline for 7 min three times, and sputum was collected and pooled after each inhalation. Spirometric evaluations were performed before and after each inhalation during induction (Vitalograph S-model Vitalograph Ltd., Buckingham, England at regular visits in the steady state, EasyOne model 2001 Ndd Medizintechnik AG, Zurich, Switzerland at exacerbation visits). Sputum samples were kept on ice until undergoing quality control less than 30 min after sampling. For the sputum samples to be considered of acceptable quality there had to be >1 million/mL cells, <20% epithelial cells and the leucocyte viability had to be >30%. If the samples were of sufficient quality, they were further treated by standard protocol [7] to separate the supernatant from the cell pellet. All materials were aliqouted and frozen at −80 °C.

### DNA extraction and 16S rRNA sequencing

The samples were thawed and treated with sputasol (Oxoid). They underwent pre-lysis with Lysostaphin (4000 U/mL), Lysozyme (10 mg/mL) and Mutanolysin (25,000 U/mL) (Sigma-Aldrich). To avoid shearing of free DNA each sample was centrifuged and supernatants and pellets separated. The pellets underwent mechanical and chemical lysis using the FastPrep-24 Instrument and reagents from the FastDNA Spin Kit (MP Biomedicals, LLC, Solon, OH, USA). Lysates and supernatants from each sample were recombined and the extracted DNA was further purified using the FastDNA Spin Kit. Library preparation and sequencing of the V3-V4 region of the 16S rRNA gene was carried out according to the protocol for Metagenomic Sequencing Library Preparation for the Illumina Miseq System (Part # 15044223 Rev. B, MiSeq Reagent Kit v3). Amplicon PCR was carried out with a total of 45 cycles and followed by Index PCR using primers from the Nextera XT Index Kit (Illumina Inc., San Diego. CA, USA). Pooled, normalized samples went through 2 × 300 cycles of paired-end sequencing. Each of the sample pairs were processed on the same day, and for all pairs we used the same reagent kits throughout DNA extraction, PCR and sequencing.

### Bioinformatics analyses

FASTQ-files were computed using Quantitative Insights Into Microbial Ecology (QIIME) v.1.9.1 [9, 10]. First, forward and reverse reads were assembled, after which sequences that did not pass quality demands as advised by QIIME were removed [11]. The accepted sequences were clustered into operational taxonomic units (OTUs) through open reference OTU-picking using uclust v.1.2.22 [12] and the GreenGenes Database v.13_08 [13]. The latter was also used for taxonomic assignment with analyses performed on GreenGenes taxonomic level 6 (genus). The clustering was based on sequence similarity with a threshold of 97%, which is considered the conventional cut-off for 16S rRNA maker-gene surveys and representative for bacterial species [14]. For each OTU a representative sequence was aligned using PyNAST v.1.2.2 [15], and sequences not successfully aligned were omitted from further analyses. A phylogenetic tree was built using FastTree v.2.1.3 [16]. Counts of observations (OTUs) on a per-sample basis were stored in Biological Observation Matrix (BIOM) format and OTUs containing less than 0.005% of the total number of sequences were removed according to QIIME guidelines [10, 11].

### Statistical analyses

Comparisons of the taxonomic distribution between pairs were performed both by calculating the Yue-Clayton measure of dissimilarity (1-θ_YC_ - range 0 to 1; 0 indicates perfect similarity, 1 perfect dissimilarity) [17], and using limits of agreement (LOA) calculated from Bland-Altman plots [18, 19]. Both methods allow evaluation of quantitative differences within each pair.

The mean number of sequences allocated to each identified taxa in the 36 ISS was compared to that found in the 36 SSS, using log-likelihood ratio tests with Bonferroni corrected *p*-values due to multiple comparisons. The comparisons were made between samples normalized through rarefaction with random subsampling without replacement. Comparisons of alpha- and beta-diversity were performed on rarefied OTU-tables [20] with available statistical analyses incorporated in QIIME-scripts. Alpha-diversity (within-sample diversity) was estimated using Faith’s phylogenetic diversity, Chao1 and counts of observed OTUs. Beta-diversity is a measure of diversity between samples. To evaluate differences in phylogenetic, quantitative beta-diversity pair-wise, weighted UniFrac (WUF) significance tests were applied [21]. All 72 samples were compared generating 2556 comparisons, for which Bonferroni corrected *p*-values were used.

Principal coordinates analysis (PCoA)-plots of WUF distances between sampling methods were used for visualization of distances in three-dimensional space using Procrustes analyses and transformations of principal coordinates 1-3 [22]. Analyses of similarities (ANOSIM), were used to compare differences in beta-diversity between ISS and SSS when samples were grouped by type [23], both considering WUF and its qualitative equivalent unweighted UniFrac distances (UWUF).

Stata 13.1 (StataCorp LP. 2013. College Station, TX) was used for generation of the Bland-Altman plots.

All relevant data were deposited at the Dryad Digital Repository (www.datadryad.org) and are referenced in the text using the following doi: http://dx.doi.org/10.5061/dryad.5gc82.

## Results

We obtained a total of 36 high-quality pairs of sputum from 30 different COPD patients. Eleven patients were women; two thirds of patients were aged 55-64 years at inclusion. Patient characteristics are summarized in Table 1.

**Table 1:** Patient characteristics

	n (%)
Sex	
Women	11 (37%)
Men	19 (63%)
Age	
40-54 years	4 (13%)
55 - 64 years	18 (60%)
65 - 75 years	8 (27%)
Body composition	
Normal	21 (70%)
Obese	4 (13%)
Cachectic	5 (17%)
Smoking	
Ex	18 (60%)
Current	12 (40%)
GOLD stage	
II (FEV_1_ 50-80%)	14 (47%)
III (FEV_1_ 30-50%)	12 (40%)
IV (FEV_1_ < 30%)	4 (13%)
Frequent exacerbator^a^	
No	20 (67%)
Yes	10 (33%)
Using inhaled steroids	
No	6 (20%)
Yes	24 (80%)
Using antibiotics^b^	
No	30 (100%)
Yes	0 (0%)

^a^>1 exacerbation last 12 months prior to inclusion

^b^At time of sampling

After processing of the raw data, 1004 different OTUs were identified with 2.5 of 4 million sequences belonging to samples delivered at exacerbations (25 of the 36 sputum pairs).

### Taxonomy

The 1004 OTUs identified by QIIME were sorted into 106 different taxa by QIIME’s taxonomic summary command. First, we calculated the Yue-Clayton measure of dissimilarity between the mean abundances of the most dominating OTUs (each containing ≥1% of all sequences) assigned to 11 different taxa in all ISS versus all SSS. This represents a group comparison and not a pair by pair comparison. The samples were then sorted with regards to disease state at time of sampling. The dissimilarity (1-θ_YC_) measure was 0.04 when disease state was not considered. For exacerbation samples the dissimilarity (1-θ_YC_) measure was also 0.04, and for stable state samples 0.03. Performing the same analyses including also low-abundance OTUs gave a dissimilarity (1-θ_YC_) measure of 0.04 when all samples were included, and 1-θ_YC_ of 0.04, and 0.05 for exacerbation and stable samples respectively.

**Fig. 1:**
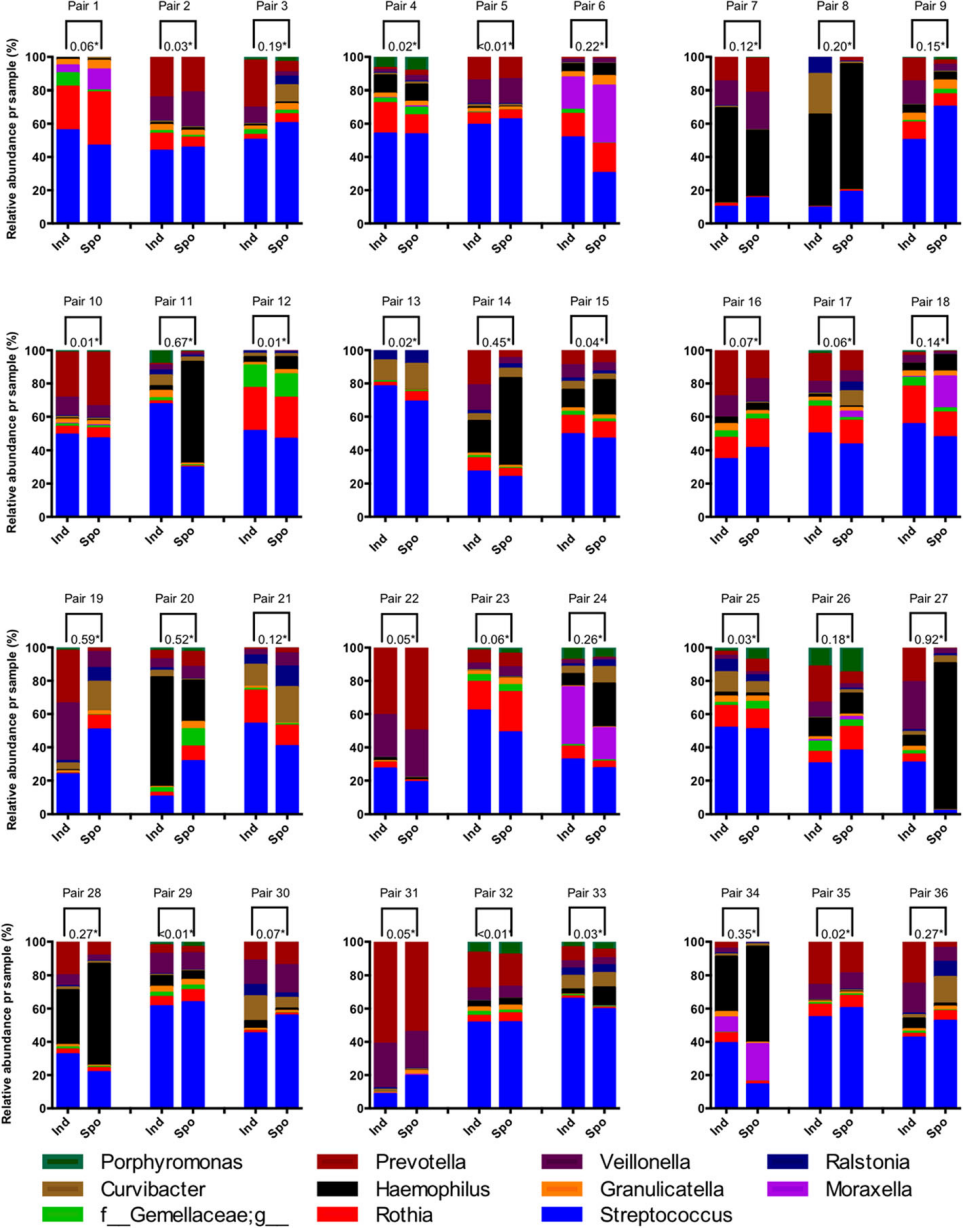
Compositional taxonomic differences for each sample pair** on genus level for the 11 most dominating taxa***. * Yue-Clayton dissimilarity = Range 0-1. **Pair 1-11: Stable state Pair 12-36: Exacerbation. ***OTUs containing <1% of sequences were omitted from the data before performing taxonomic summaries (GreenGenes database level 6 = genus)

Taxonomic compositional differences within sample pairs were visualized as bar graphs for the same 11 dominating taxa (Fig. 1). As shown, there were obvious visual differences within some pairs. 1-θ_YC_ was calculated both for dominating OTUs exclusively, and for all OTUs. When evaluating dissimilarities pair-wise for dominating OTUs and their associated taxa, 1-θ_YC_ ranged from <0.01 – 0.92 (Fig. 1). 1-θ_YC_ ranged from <0.01 – 0.58 when also including sparse OTUs and corresponding taxa (data not shown). With 0.2 as limit for acceptable within-pair 1-θ_YC_, seven pairs were found dissimilar regardless of OTU-abundance (pairs 8, 11, 19, 20, 27, 34 and 36), while four pairs were found dissimilar only if filtering out low-abundance OTUs (pairs 6, 14, 24 and 28) or keeping low-abundance OTUs respectively (pairs 3, 13, 22 and 26).

**Fig. 2:**
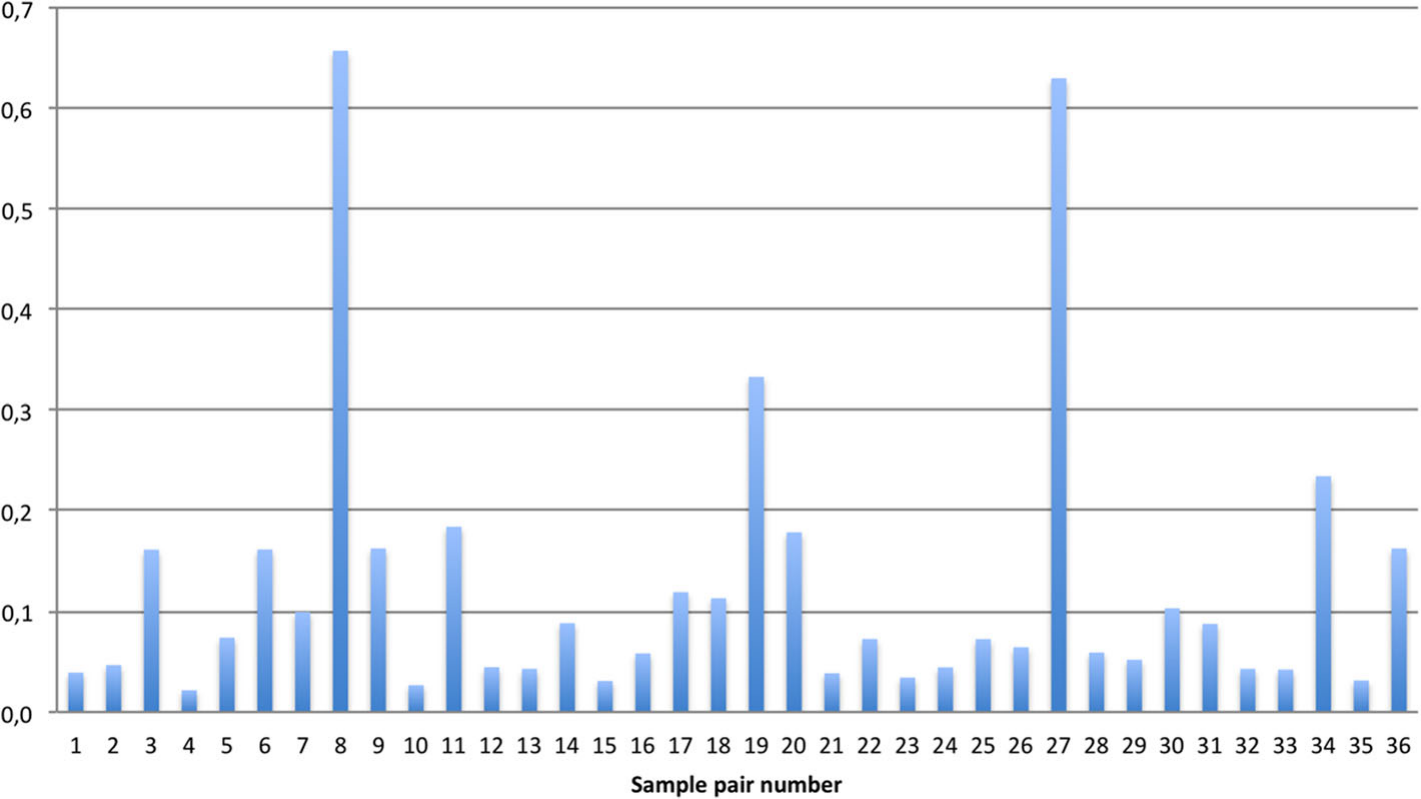
Expected differences between relative abundances of taxa per sample pair with 95% Bland-Altman limits of agreement. *Range = 0-1. **Pair 1-11: Stable state Pair 12-36: Exacerbation

To further assess differences in taxonomy between sample pairs, one Bland-Altman plot of the relative abundances of our 106 taxa was generated for each pair. From the upper and lower 95% LOA, the range is calculated (upper-lower/100) corresponding to a number between 0 and 1, where 0 indicates perfect agreement. Using this approach, we found ranges in LOA between 0.02-0.66 (Fig. 2). Setting an acceptable limit for LOA at 0.1 allows the relative abundance in each taxa to vary from ISS to SSS by 10%. With this limit 13 pairs could not be accepted as equal, including the seven pairs found too different by 1-θ_YC_ regardless of OTU-abundance (Fig. 2).

There were significant differences between the mean abundances in induced versus spontaneous samples for 15 taxa in either the exacerbated or the stable state (Table 2). For instance for the well known pathogenic *Moraxella*, there were almost twice as many sequences in all spontaneous samples compared with all the induced samples both during exacerbations and in the stable state (*p* < 0.001, Table 2). Also *Haemophilus* was consistently more abundant in spontaneous than in induced samples.

**Table 2:** Mean number of sequences per sample constituting the 15 taxa^a^ found in significantly different amounts in induced and spontaneous sputum from COPD patients with and without respect to disease state

	All samples			Exacerbation			Stable state		
Taxonomy^b^	Induced	Spontaneous	p^c^	Induced	Spontaneous	p^c^	Induced	Spontaneous	p^c^
f_Prevotellaceae;g_Prevotella	2499.2	1760.3	<0.001	2633.1	1817.1	<0.001	2349.5	1763.0	<0.001
f_Pasteurellaceae;g_Haemophilus	1471.9	2440.3	<0.001	1308.2	2356.6	<0.001	1957.7	2859.8	<0.001
f_Moraxellaceae;g_Moraxella	259.2	542.3	<0.001	234.4	456.5	<0.001	353.9	798.0	0.001
f_Veillonellaceae;g_Veillonella	1488.3	1170.8	<0.001	1618.8	1173.1	<0.001			
f_Veillonellaceae;g_Megasphaera	179.9	100.0	<0.001	201.9	111.5	<0.001	134.8	79.4	0.01
f_Corynebacteriaceae;g_Corynebacterium	44.9	12.0	<0.001	60.3	14.9	<0.001			
f_Oxalobacteraceae;g_Ralstonia				423.3	604.7	<0.001	331.8	213.3	<0.001
f_Comamonadaceae;g_Curvibacter				518.7	713.0	<0.001	430.7	269.5	<0.001
f_Leptotrichiaceae;g_Leptotrichia				171.8	95.2	<0.001			
f_Neisseriaceae;g_Neisseria							344.5	149.8	<0.001
f_Gemellaceae;g_Gemella							84.9	16.5	<0.001
f_Gemellaceae;g_							452.7	273.6	<0.001
f_Neisseriaceae;g_							79.5	17.5	<0.001
f_Leptotrichiaceae;g_Leptotrichia							123.6	217.6	<0.001
f_Actinomycetaceae;g_Actinomyces							350.1	256.0	0.001

^a^Rarefied OTU-tables: Sequences/Sample = 18,250 for All samples and Exacerbations, for Stable state: 19,743

^b^GreenGenes Level 6: f_ = Name of family level g_ = Name of genus level. One hundred six different taxa in total

^c^log-likelihood ratio test, Bonferroni corrected due to multiple comparisons

### Diversity

No statistically significant differences were found in alpha-diversity (Table 3).

**Table 3:** Mean within sample diversity (alpha diversity) in induced versus spontaneous sputum in COPD by different alpha diversity indices

	All samples			Exacerbation			Stable state		
Diversity Indices	Induced	Spontaneous	p^a^	Induced	Spontaneous	p^a^	Induced	Spontaneous	p^a^
Faith’s Phylogenetic Diversity									
mean (std)	56.9 (9.3)	56.2 (8.6)	0.7	57.3 (9.5)	56.2 (8.8)	0.7	57.1 (8.5)	56.6 (8.4)	0.9
Chao1									
mean (std)	646.5 (116)	638.3 (107.5)	0.7	655.0 (118.5)	642.1 (105.1)	0.7	640.8 (110.4)	643.6 (105.3)	0.9
Observed OTUs									
mean (std)	543.2 (104.9)	528.8 (106.1)	0.6	552.9 (105)	534.3 (107.1)	0.6	531.6 (102.4)	527.7 (103.2)	0.9

^a^Non-parametric two-sample t-test using Monte Carlo permutations

However, we found statistically significant differences (*p* < 0.01, Bonferroni corrected due to multiple comparisons) in the pair-wise quantitative, phylogenetic beta-diversity as evaluated by weighted UniFrac for 9 pairs (Pair 3, 14, 17, 19, 26, 30, 32, 33 and 36).

**Fig. 3:**
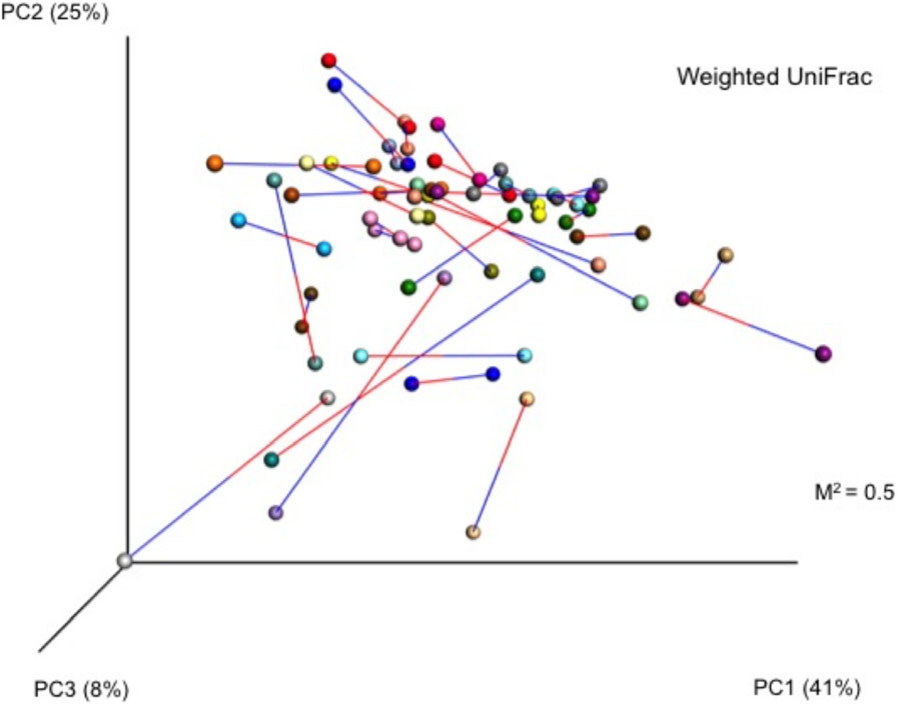
PCoA plots modeling multi-dimentional distribution of OTUs derived from induced and spontaneous sputum samples*. *Sample pairs connected by bars - blue bars attach to induced sputum samples, red bars attach to spontaneous sputum samples

The principal coordinates analysis (PCoA) plots are presented in Fig. 3.

Each dot represents the weighted UniFrac distance diversity measure for each sample, and lines illustrating the distance between paired sputum are shown (Blue line attaches to ISS, red to SSS). The greater the distance, the greater is the difference. Although this is a two-dimensional visualization of a three-dimensional calculation, Fig. 3 clearly shows that the distances between paired samples varied. A Monte Carlo simulation with 1000 permutations was applied giving M^2^ = 0.5, confirming the visual interpretation (Identical plots: M^2^ = 0, if completely dissimilar M^2^ = 1).

Using analyses of similarities (ANOSIM), we found no significant differences in means of beta-diversity (UWUF and WUF) between ISS and SSS when samples were grouped by type. This was true both in stable state and at exacerbations (*p* > 0.05).

## Discussion

This study on sputum samples collected sequentially using two different methodologies from COPD patients and treated equally by the same protocol shows that in approximately 1/3 of sputum pairs either taxonomical and/or diversity analyses differ significantly. Discordance between induced and spontaneous samples were seen both at exacerbations and during stable state.

The strength of the current study is the unique data material; including induced and spontaneous sputum samples collected simultaneously, treated by the same protocol [7, 8], both at the stable state and during exacerbations. However, there are some methodological issues to discuss. First, after either induction or through sampling of spontaneous sputum, sputum was kept in a clean collection dish, and material selected by trained technicians for further analyses. This is the standard approach [24], but entails a natural variation of sample selection. However, there is no reason to believe the judgment of the technician should differ between sample types, and all other processing was the same for both types of sputum.

Second, errors may occur during DNA extraction, PCR or sequencing steps. All pairs were run simultaneously for all steps in the laboratory protocol, including on the same flowcell in the Illumina MiSeq. However, random errors could be a factor, and based on the plots of the dominant taxa in Fig. 1, we chose the seven most visually dissimilar pairs (pairs 11, 14, 19, 20, 26, 27 and 36) and three visually similar pairs (pairs 2, 5 and 29) and redid the laboratory analyses. For only one of the 20 samples (pair 26, ISS) were the results convincingly different visually from the first to the second run. Since this was not a random selection, the likely error is much lower than 5%, and we do not believe our results are due to random laboratory error. For data analyses we chose to keep the sequences from run two for the ten re-run pairs.

Third, low biomass samples are prone to contamination from multiple sources during laboratory handling [25]. Approaches to handle the potential contamination include sequencing of known (“mock”) communities, negative control samples, and manual curation of the sequencing output. A potential contaminator in our study is the saline used for induction. Unfortunately it was not stored at the time the procedures were performed, and so an important limitation to the current study is that we were not been able to examine the influence of negative saline controls on our samples. All samples were treated exactly similar at all steps of analyses, thus minimizing confounding from potential contamination. However, as the biomass and dilution of each sample in a pair may differ, we cannot exclude that samples could be differentially affected by contamination from saline. Finally, as in other studies comparing sampling methods’ impact on microbiota [5, 26, 27], the number of samples is limited, and the statistical power therefore reduced.

One of the challenges in microbiome research is that the technological advancements develop faster than the establishment of statistical tools to assess results. What signifies a true compositional difference between two supposedly similar samples where each contains a large number of relative abundances of sequences is still an unsettled question. The cut-off for the two indices used for assessment of taxonomic differences, <0.2 for the Yue-Clayton dissimilarity index (1-θ_YC_) and <0.1 for LOA from the Bland Altman plots, are arbitrary, and no established consensus regarding these values exist. Similar for the Procrustes M^2^ value there are no defined limit [28].

Finally, what constitutes a true clinically important difference is also an unsettled question. It could be that the entire ecological content of a sample is more relevant for disease, or it could be the presence of a few, perhaps even only one, low-abundant pathogen. If the latter is true, a cut off <0.2 for 1-θ_YC_ and <0.1 for LOA will be too crude. With a sample size of 36 sputum pairs, this study did not have the power to evaluate whether ISS or SSS better correlated with clinical data. Future studies with larger sample sizes are needed to elucidate this question.

This study brings forward new information on the much used sputum samples in studies on COPD patients. Pair-wise comparisons of taxonomic composition on genus-level between ISS and SSS from lung patients have not previously been done to our knowledge. Neither have comparisons of alpha- or beta-diversity between ISS and SSS earlier been reported. Induced sputum sampling is an established protocol for studying COPD patients at stable state [29]. Common for both ISS and SSS is that they sample both lungs in contrast to bronchoalveolar lavage and biopsies, and are more easily accessible material. Spontaneous sputum is easier to collect during AECOPD when sputum production increase, and may be preferred by some for fear that induction may worsen airway obstruction. However, we have previously shown that induction can safely be performed during COPD exacerbations, at least with up to 3% hypertonic saline [7].

There are potential reasons why spontaneous and induced sputum samples would differ in their microbial content. Different airways regions have been shown to harbor different communities [30, 31], possibly partly due to different ventilation-circulation ratios in the lower and upper parts of the lungs, and possibly due to differences between proximal and distal airways. It has been shown before that sputum sampled early during induction has a different composition of cells than sputum sampled late during induction [32, 33]. Spontaneous samples may resemble proximal airways more than the distal sampled by induction, and possible differ in ability to sample upper and lower airways.

Abundant OTUs and correspondingly dominating taxa in different environments have been shown to be particularly important in their habitats [34]. However, sparse members of the microbiota have also been found to contribute in pathogenic processes in the lungs [35, 36]. With this in mind we chose to examine the identified taxa emphasizing both dominating and sparse OTUs. The group comparison of mean abundances of taxa by Yue-Clayton dissimilarity showed that pooling of observations can hide differences seen between individual sample pairs.

The strength of the 1-θ_YC_ index is that it measures structural dissimilarity by calculating the proportions of both shared and unshared components in a community [17]. The number of pairs where ISS and SSS were considered too dissimilar to be accepted as good substitutes for each other (1-θ_YC_ > 0.2) was the same regardless of focusing on taxonomic assignment of only abundant OTUs or accepting all OTUs. In both cases 1 of 3 pairs would render different results depending on which sample type was picked to represent the patient.

The Bland-Altman’s LOA analyses confirmed the findings using Yue-Clayton’s dissimilarity, in that ISS and SSS did not provide the same results in a significant fraction (13 of 36) of pairs when evaluating taxonomical composition in sputum from COPD patients.

Summarizing our findings on GreenGenes genus-level left 106 unique taxa. When comparing the mean abundance of sequences in each taxa between sample types, 8.5% of taxa were found in statistically significant different levels between sputum types during exacerbations, and 11.3% in the stable state. Both of the known potential pathogens *Haemophilus* and *Moraxella* were significantly more abundant in spontaneous samples compared with induced samples, both in the stable state and during exacerbations. In this new era, where the whole composition of a microbiome may be relevant for disease, it may be that induced sputum samples better reflect presence of low-abundant species in the distal airways, which are masked by frequent colonization of genera like *Haemophilus* and *Moraxella* in spontaneous samples. However, presence of both *Haemophilus* [37, 38] and *Moraxella* [39] in stable state sputum samples have shown similar higher levels of inflammatory markers in the sputum samples indicating stimulation of the immune system. Thus either sampling method may have important value in research, but important differences in interpretation of the microbiota could result from using the sputum types interchangeably.

We could not find differences in alpha-diversity between sample types. This should perhaps not be surprising considering the shared route of delivery through the oral cavity and the samples not discriminating between right and left airways. It has been shown that diversity in sputum is higher than in explant lung samples, likely due to oral contamination [30].

There were no significant differences in mean phylogenetic beta-diversity between ISS and SSS, neither when considering absence/presence data, nor when emphasizing abundances (UWUF/WUF). However, when considering samples pair-wise we found differences in WUF in 1 of 4 pairs and for UWUF differences were found in 50% of the sputum pairs. With focus on quantitative data Procrustes transformation of PCoA-plots of WUF distances this pair-wise difference was confirmed, as the distances in multidimensional space were too large to ignore for several pairs. A defined limit for Procrustes M^2^ to be considered too high to claim similarity does not exist, but levels >0.3 is indicative of influential differences.

## Conclusions

In this study we found clear discrepancies in both taxonomic composition and beta-diversity between ISS and SSS collected concurrently from COPD patients in the stable state and during exacerbations when comparing samples pair-wise. For grouped analyses the differences were subtler, potentially masking important differences. The most prudent approach in studies using sputum for microbiota analyses is to only rely on either induced or spontaneous sputum. We advise that sampling method is always reported, and that comparisons are made and presented, if both sample types are used.

## Abbreviations

AECOPD: Acute exacerbation of COPD
BCCS: Bergen COPD cohort study
BCES: Bergen COPD exacerbation study
CF: Cystic fibrosis
COPD: Chronic obstructive pulmonary disease
ISS: Induced sputum samples
LOA: Limits of agreement
OTU: Operational taxonomic unit
PCoA: Principal coordinates analysis
QIIME: Quantitative insights into microbial ecology
SSS: Spontaneous sputum samples
UFUW: Unweighted unifrac
WUF: Weighted unifrac
